# Supporting patient choice in IOL selection

**Published:** 2026-08-12

**Authors:** Shahir Umar Bello, Daksha Patel

**Affiliations:** 1 Consultant Ophthalmologist/Lecturer: Abubakar Tafawa Balewa University (ATBU) and ATBU Teaching Hospital, Bauchi, Nigeria.; 2 Associate Professor and Fellow: Centre of Online and Distance Education, University of London, UK.

**Some patients may wish to retain good near vision after cataract surgery. What are their options, and how can we support them?**

**Figure:**
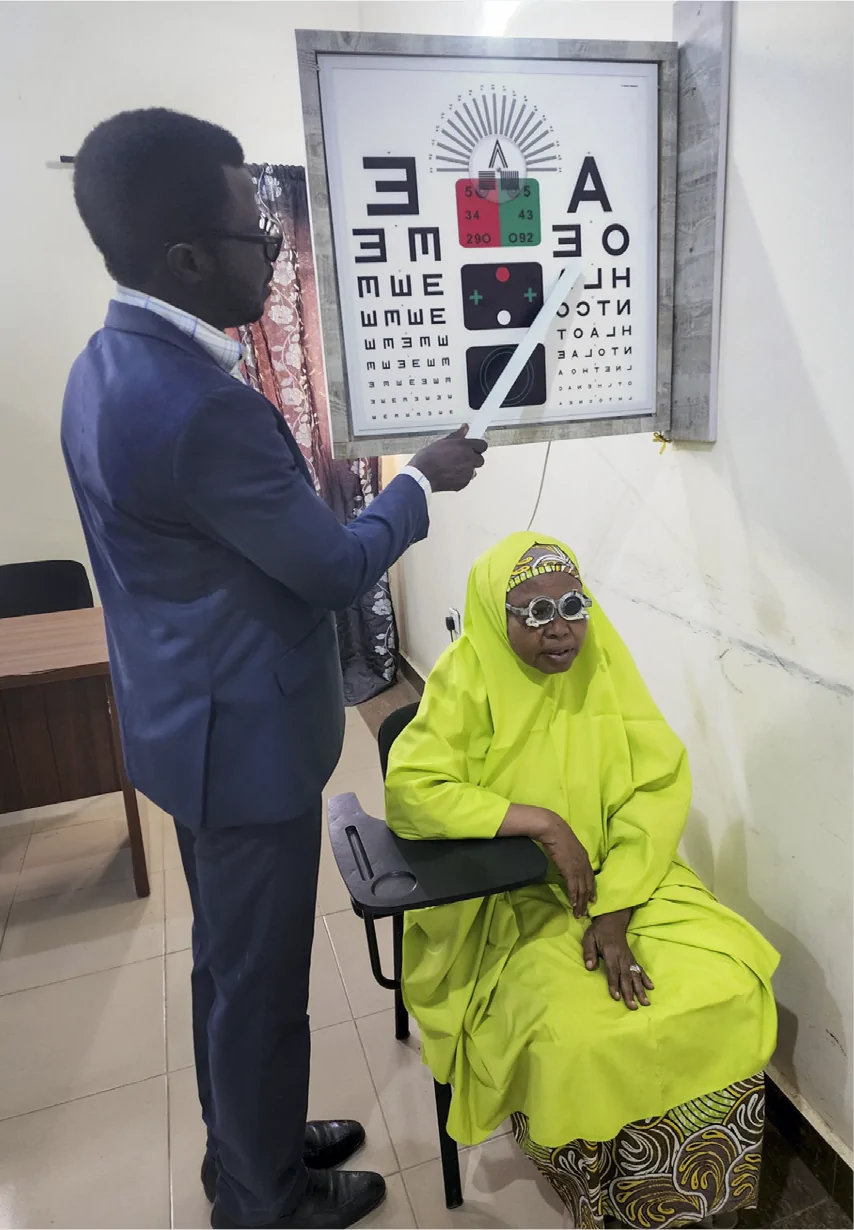
Postoperative distance and near visual acuity testing, followed by refraction and spectacle dispensing, if needed, are vital to ensure good visual outcomes for patients after cataract surgery. NIGERIA

© SHAHIR UMAR BELLO CC BY-NC-SA 4.0

The standard approach to intraocular lens (IOL) selection is to use monofocal IOLs – the most affordable and widely available type – to correct for distance vision, usually aiming for distance visual acuity (VA) of 6/6. This makes sense, generally speaking, as the quality of cataract surgery is defined in terms of distance visual acuity. In fact, the current WHO standard for cataract surgery is ‘effective cataract surgical coverage’, defined as the proportion of patients needing cataract surgery who have received surgery and have distance VA of 6/12 or better, which is the cut-off for driving in most countries.

Monofocal IOLs provide clear vision at a specific focal distance. Most patients who receive monofocal IOLs selected to provide good distance vision (emmetropia) will therefore need to purchase near-vision spectacles in order to use a computer or a smartphone, or to undertake detailed near work.

While distance vision remains important for the majority of patients, providing patient-centred care means acknowledging each patient as an individual, with individual needs. Some patients may prioritise good near vision over good distance vision. This is especially true for many patients with myopia, who are used to being able to see well up close without the need for near-vision spectacles. Some patients may also wish to avoid using near-vision (‘reading’) spectacles for cosmetic or other reasons.

## Discussing options with the patient

### 1. Assess the patient: their vision, health, and lifestyle

Assess the patient's vision with and without correction, their ocular symptoms, and previous eye procedures.

To better understand the patient's lifestyle and vision needs, ask about their work and leisure activities, and how they need and want to use their sight: would this mainly be for long distances, middle distances, or near distances? Are there things they would like to do, or things they can no longer do which they would like to resume?

Remember to allow time for the patient to ask questions.

### 2. Ask about their history and preferences

What is their history of spectacle use, and their willingness to keep wearing spectacles, or switch from distance to near-vision spectacles? Patients with myopia sometimes prefer to maintain some degree of shortsightedness, as they have been used to reading or using a smartphone without the need for spectacles.

Find out how the patient would feel about changing the type of spectacles they will need after surgery. Explain that, if they opt for better distance correction without spectacles, they may need spectacles for near vision, and vice versa.

### 3. Inform the patient about their options

Explain the various options available (see panel). The choice depends on the patient's visual requirements and lifestyle; for example, whether they depend on near vision for their job or wish to avoid wearing spectacles altogether after cataract surgery.

### 4. Support them to make a decision

There may not be a single definitive solution to a patient's issue. We recommend shared decision-making: an approach in which health care providers and patients collaboratively discuss treatment options to select the most appropriate one, depending on the patient's needs and preferences.

Explain all the costs involved and support the patient to work out whether they can afford the direct costs associated with the procedure, including insurance coverage (and the cost of premium lenses, if those are selected) and the cost of any spectacles that may be needed after surgery.

In some contexts, decisions regarding surgery for elderly parents are made collectively by the family. Because it is often the patient's children who will pay for surgery, it is important to explain to them that visual impairment is not a natural consequence of ageing and that cataract surgery can significantly improve the person's visual function, restore their independence, and improve their quality of life.

### 5. Counselling about postoperative care and refraction

Explain to patients how important it is that they come back for postoperative follow-up and refraction, so that they can achieve the best possible final visual outcome.

See the article on page 8 for further postoperative counselling guidance.

*To read the full version of this article, including a case study, visit* https://cehjournal.org/articles/10.56920/cehj.865

**Intraocular lens options for patients who want to retain some near vision**

**Figure:**
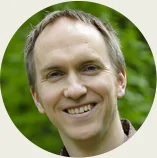
John Buchan Programme Director: MSc Public Health for Eye Care, International Centre for Eye Health, London School of Hygiene & Tropical Medicine and Clinical Lead: RCOphth National Ophthalmology Database Cataract Audit, Leeds, UK.

**Figure:**
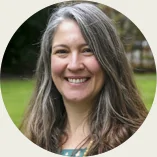
Elmien Wolvaardt Editor-in-Chief: Community Eye Health Journal, International Centre for Eye Health, London School of Hygiene & Tropical Medicine, UK.

Discussing refractive expectations from cataract surgery is necessary for most patients, but particularly for myopes. All their life they have seen near objects clearly. If lens implants are selected targeting good distance vision (emmetropia) they will lose this ability, and will need near-vision spectacles. For these patients, or others who prioritise good near vision, there are various options.

**Option 1: Choosing IOLs that leave the patient slightly short-sighted**

Standard monofocal IOLs can be selected to leave a patient slightly under-corrected for distance vision. If a patient is intentionally left slightly short-sighted, they will have the benefit of having reasonable vision for distance, but will also be able to focus on some near objects or tasks.

**Who it is useful for:** Patients who already have myopia and are used to wearing spectacles for distance vision (and will continue to be able to afford them). These patients may prefer to retain good uncorrected near vision.

**What to say to patients:** Explain to patients what they are likely to be able to see nearby and in the distance, depending on the level of myopia aimed for (see Table 1 for some examples).

Aiming to leave a patient with myopia of −1.00 D to −1.50 D will allow them to focus at 1 metre to 67 cm, with VA of 6/18 to 6/30 (see Table 1), provided their eyes are otherwise healthy. These patients will still need near-vision spectacles for detailed tasks nearer than 67 cm, and distance vision spectacles for driving. If a patient wants to be able to focus on tasks at distances of 50 cm or 33 cm, without the need for near-vision spectacles, select IOLs that will give them −2.00 D or −3.00 D myopia, respectively. The patient would need spectacles for most distance vision tasks (see Table 1).

**Option 2: Monovision**

An alternative is monovision; this involves using standard monofocal IOLs of different powers, calculated to correct the dominant eye for distance vision and the non-dominant eye for near vision. Some patients find that this works well for them, and they have the benefit of not having to wear spectacles at all. Others may not be able to easily adjust to monovision, particularly if the difference between the two eyes is greater than 2.00 D.

**Top tip:** Aiming for a difference of as little as 1.00 D between the two eyes would give some useful near vision as well as good distance vision. For example, aiming −0.25 D in the first eye and −1.25 D in the second eye will be almost universally tolerated and give good distance vision, as well as near vision focused as close as 80 cm.

**Who it is useful for:** Consider for patients undergoing cataract surgery in both eyes, particularly those who are unlikely to purchase spectacles after surgery. Patients who naturally have one eye focused for near and one for distance, and those who have tried monovision contact lenses and tolerated them, may find it easier to adjust to monovision.

**NOTE:** People who are very likely to obtain prescription spectacles postoperatively may not need this, particularly if they have a strong reason to want good unaided vision at a particular distance (e.g. if they spend the majority of their time driving, or doing detailed near work).

**Option 3: Multifocal and other specialist lenses**

In some centres, multifocal IOLs may be available. They are designed to give good near and distance vision, but they may fall short of expectations and cause side-effects such as glare; they can also be very expensive.

**What it involves:** Multifocal intraocular lenses split light into multiple focal points, allowing patients to see objects in focus at near, intermediate and far distances.

**Who it is useful for:** Those who can afford the technology and who don't want to wear spectacles.

**NOTE:** These lenses are not suitable for people who need to drive after dark (as the lenses can cause haloes around lights at night) and people with conditions such as age-related macular degeneration, diabetic retinopathy, and glaucoma, as the lenses can make it more difficult to examine the back of the eye.

**Table 1:** Some suggestions for explaining the impact of IOL choice on near and distance vision.

Targeted degree of myopia	Near working distance	Distance VA	Near Vision	Distance Vision
**−1.00 D to −1.50 D**	1 m to 67 cm	6/18 to 6/30	You will be able to prepare food, count money, or do agricultural work like weeding or harvesting, without needing near-vision spectacles.	You will need distance vision spectacles in order to drive, or to clearly see the face of someone on the other side of the street.
**−2.00 D**	50 cm	6/45	You will be able to read books and newspapers, or write messages on a phone, without needing near-vision spectacles.	You will need distance vision spectacles to recognise the faces of people you know if they are more than 1–2 metres away from you.
**−3.00 D**	33 cm	6/120	You will be able to read food labels or medication pamphlets, or do very fine or detailed needlework, without needing near-vision spectacles.	Without distance vision spectacles, your eyesight will be so limited that you may have difficulty moving around safely, even at home.

